# Functional characterization of protein domains common to animal viruses and mouse

**DOI:** 10.1186/1471-2164-12-S3-S21

**Published:** 2011-11-30

**Authors:** Akira R Kinjo, Yutaro Kumagai, Huy Dinh, Osamu Takeuchi, Daron M Standley

**Affiliations:** 1 Institute for Protein Research, Osaka University, 3-2 Yamadaoka, Suita, Osaka 565-0871, Japan; 2 Laboratory of Host Defense, WPI Immunology Frontier Research Center (IFReC), Osaka University, 3-1 Yamadaoka, Suita, Osaka 565-0871, Japan; 3 Laboratory of Systems Immunology, WPI Immunology Frontier Research Center (IFReC), Osaka University, 3-1 Yamadaoka, Suita, Osaka 565-0871, Japan

## Abstract

**Background:**

Many viruses contain genes that originate from their hosts. Some of these acquired genes give viruses the ability to interfere with host immune responses by various mechanisms. Genes of host origin that appear commonly in viruses code for proteins that span a wide range of functions, from kinases and phosphotases, to cytokines and their receptors, to ubiquitin ligases and proteases. While many important cases of such lateral gene transfer in viruses have been documented, there has yet to be a genome-wide survey of viral-encoded genes acquired from animal hosts.

**Results:**

Here we carry out such a survey in order to gain insight into the host immune system. We made the results available in the form of a web-based tool that allows viral-centered or host-centered queries to be performed (http://imm.ifrec.osaka-u.ac.jp/musvirus/). We examine the relationship between acquired genes and immune function, and compare host-virus homology with gene expression data in stimulated dendritic cells and T-cells. We found that genes whose expression changes significantly during the innate antiviral immune response had more homologs in animal virus than genes whose expression did not change or genes involved in the adaptive immune response.

**Conclusions:**

Statistics gathered from the MusVirus database support earlier reports of gene transfer from host to virus and indicate that viruses are more likely to acquire genes involved in innate antiviral immune responses than those involved in acquired immune responses.

## Background

Some viruses that infect vertebrates are able to acquire genes from their hosts[1,2]. Host organisms, in turn, have developed intricate mechanisms such as the innate and adaptive immune responses to defend themselves against viruses and other pathogens. Acquired genes that increase the chances of survival within the hosts, can thus give us a unique view of the host’s immune system. To understand the defense systems of vertebrates against viruses, comprehensive knowledge of relationships between viral and mouse proteins is necessary. Here, we select mouse (*Mus musculus*) as a model vertebrate organism, since genetic techniques are well established for mice, and they have many genes orthologous to genes in humans. We considered the overlap between entries in the conserved domain database, and homologous domains in mouse and in a wide range of viral proteins. The result of this comprehensive comparison was compiled as the MusVirus database (DB).

In order to characterize the functions of acquired domains on a biological level, we mapped each mouse entry to DNA microarray probe identifiers, allowing sets of differentially expressed genes to be uploaded to MusVirus. Here, we examined the number of viral homologs to genes differentially expressed in dendritic cells following stimulation of innate immune response pathways. For comparison we examine genes whose expression levels remained unchanged upon such stimulation, as well as genes differentially expressed upon stimulation of T cell receptors in T cells. Together, these results indicated that genes involved in innate immune response pathways were more likely to be acquired by viruses than genes involved in adaptive immune response pathways or genes whose expression levels do not change significantly upon stimulation.

## Results and discussion

**Figure 1:**
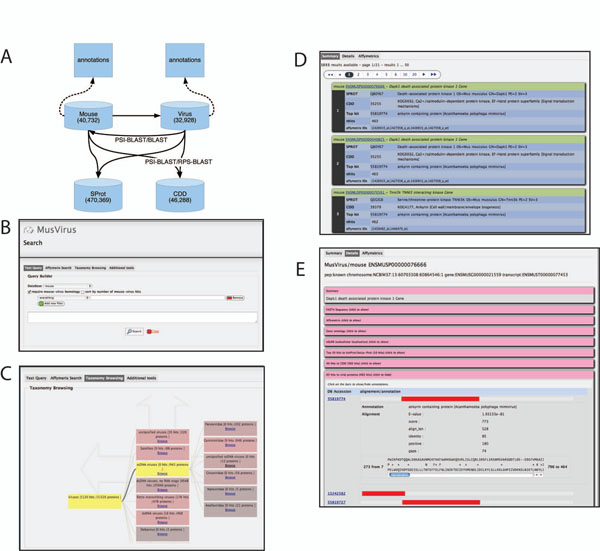
**MusVirus structure and interface.** A)The backend database contains PSI-BLAST alignments between mouse and viral proteins along with annotation and alignments to CDD and Sprot databases. B) The top page of web interface allows text-based querying. C) Graphical interface for browsing viral taxonomy; each leaf node can be used to generate a DB query for hits in mouse. D) Summary results page for a mouse-based query. E) Detailed results page shows alignments, and links to external DBs.

As described in the Methods section, the Web interface to MusVirus enables text-based keyword searches, browsing by viral taxonomy, and batch upload of microarray identifiers (Figure 1). Below, we review the results of such searches focusing in functional characterization of mouse-virus homologs.

## Overview of homologs between mouse and viral proteins

**Figure 2:**
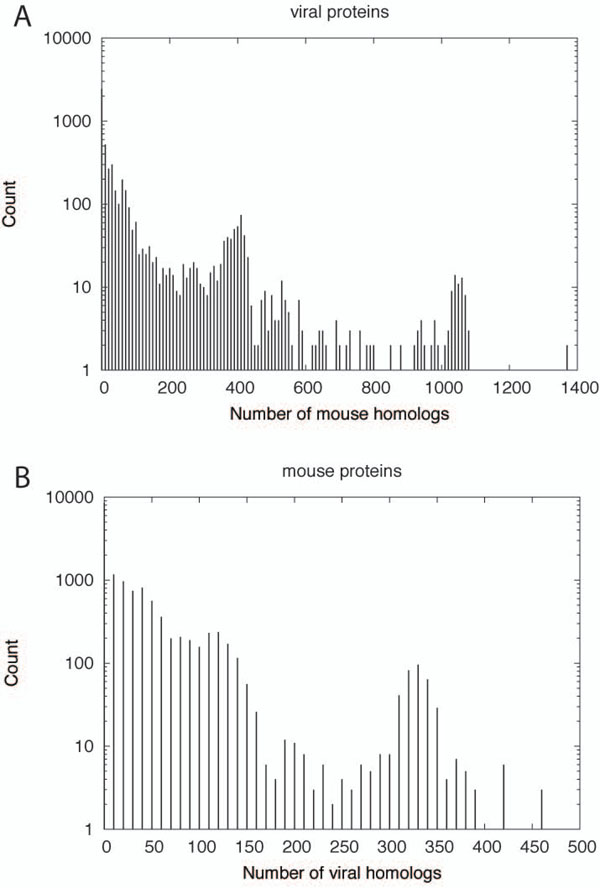
**Distribution of MouseVirus hits.** Histograms of the number of viral homologs of mouse proteins (A) and mouse homologs of viral proteins (B).

As a result of PSI-BLAST searches of mouse proteins against viral proteins, 15,127 out of 40,732 (37.1%) of mouse proteins were found to have at least one viral homolog. Among these, 1,414 proteins had more than 100 viral homologs. Mouse proteins with many viral homologs were often annotated as kinases and/or ankyrin repeats. Dapk1 (death-associated protein kinase 1, Ensembl ID: ENSMUSP00000076666), a positive mediator of interferon-induced apoptosis, had the largest number (463) of viral homologs. PSI-BLAST searches of viral proteins against mouse proteins detected 5,321 out of 32,928 (16.2%) proteins from 1,190 viral species having at least one mouse homolog (1,053 had more than 100 mouse homologs). Protein-tyrosine kinase of Y73 sarcoma virus, which causes cellular transformation leading to tumor formation [3], had the largest number (1,381) of mouse homologs. Although the fraction of viral proteins having mouse homologs is smaller than that of mouse proteins having viral homologs, the distributions of the number of homologs (Figure 2) suggest that viral proteins tend to be similar to a large number of mouse proteins if they do have mouse homologs. These results are summarized in Additional file 1.

### Shared conserved domains

After identifying homologs between mouse and viral proteins, we next asked if the similarities between homologs based on whole-sequence similarities are biased to some specific domains. To further analyze the structure of homolog similarities, we performed PSI-BLAST searches of both mouse and viral proteins against the CDD database and identified shared conserved domains. 7,222 CDD domains (out of 46,288 in total) were shared between at least one pair of mouse and viral proteins. The most frequently shared CDD domain was integrin-linked kinase (KOG0195), which has been linked to mammary tumor progression [4] and was found in 1,247 and 495 mouse and viral proteins, respectively. Out of 20 most frequently shared CDD domains, nine were kinases and eight contained ankyrin repeats. 366 CDD domains matched to more than 1,000 mouse proteins, and 86 % of these were kinases. To systematically identify the characteristics of shared conserved domains, we compared the frequencies of the words in annotations between shared CDDs and non-shared CDDs (a “non-shared CDD” is a CDD that matches either a mouse or a viral protein but not both). The words most well-representing the shared CDDs were related to serine/threonine kinases (“STKs”) and protein tyrosine kinases (“PTK,” “PTKs,” “PTKc”) followed by GT1 family of glycosyltransferases (“GT1”) and the CCX motif involved in various Rab subfamilies (“CCX”).

## Mouse-specific viruses

Among the 24 mouse-infecting viruses, 60 proteins from 13 species had at least one mouse homolog. As listed in Table 1, among the 13 species, murid herpesvirus 4 (MuHV-4) had the largest number (12) of mouse homologs, followed by Moloney murine leukemia virus and Friend murine leukemia virus. There were 8 retroviruses most of which had mouse homologs related to retrotransposon-like gene products.

**Table 1:** Distribution of mouse homologs across mouse infecting viruses

Species	Virus type	Number of homologs^1^	Total number of proteins^2^	Number of mouse homologs^3^
Murid herpesvirus 4	dsDNA	12	74	83
Moloney murine leukemia virus	retro	8	14	22
Friend murine leukemia virus	retro	6	10	14
Mouse mammary tumor virus	retro	5	14	8
Moloney murine sarcoma virus	retro	4	6	2057
Murid herpesvirus 2	dsDNA	4	8	1828
Abelson murine leukemia virus	retro	4	27	31
Murine hepatitis virus strain A59	ssRNA	4	27	9
Murine hepatitis virus strain JHM	ssRNA	4	167	112
Murine osteosarcoma virus	retro	3	5	28
Murid herpesvirus 1	dsDNA	2	2	5
Murine type C retrovirus	retro	2	3	9
Rauscher murine leukemia virus	retro	2	161	41

^1^The number of viral proteins that have some homologs in mouse. ^2^The total number of proteins in the virus (found in the database). ^3^The total number of mouse proteins homologous to some viral proteins.

We present a summary of results for MuHV-4 in Table 2. On the one hand, some proteins such as the large tegument protein matched mouse proteins at relatively low-complexity regions. Although there may be biological meaning of these matches, their interpretation requires careful examination. On the other hand, proteins such as membrane protein G74 (homologous to chemokine receptors) and complement control proteins (homologous to the mouse equivalent) are known to interfere with the mouse immune system [5,6]. Other proteins such as tegument serine/threonine protein kinase [7], cyclin and E3 ubiquitin ligase MIR1 may be used to hijack the mouse signal transduction system, as discussed below.

**Table 2:** Murid herpesvirus 4 proteins with mouse homologs.

Protein ID^1^	Protein name	Number of mouse homologs
9629556	complement control protein	41
9629601	E3 ubiquitin ligase MIR1	17
9629628	cyclin	7
9629613	uracil-DNA glycosylase	3
9629560	DNA polymerase catalytic subunit	3
9629631	protein G75B	2
9629630	tegument protein G75C	2
9629622	ribonucleotide reductase subunit 2	2
9629632	protein G75A	2
9629577	tegument serine/threonine protein kinase	2
9629596	membrane protein G74	1
9629623	ribonucleotide reductase large	1

^1^NCBI GI number for the protein sequence

## Genes induced by innate immune responses abundant in MusVirus DB

As discussed above, MusVirus contains proteins with various biochemical annotations. We next asked whether MusVirus hits are related by their biological functions as well. Since database annotations of biological function are less complete than those of biochemical functions, we grouped proteins by their corresponding gene expression levels in stimulated immune cells. Specifically, we examined expression levels of genes induced in the innate immune and adaptive immune responses. Animal hosts provoke pleiotropic immune responses to limit viral dissemination when infected with viruses. In the course of the response, many genes are induced, not only as mediators of antiviral signaling such as type I IFN genes and proinflammatory cytokines, but also cell-intrinsic effectors such as GTPase Mx1, RNase Isg20, the SAM domain protein viperin, and the zinc finger protein ZAP1 [8]. As shown above, and discussed elsewhere, viruses have many proteins homologous to such host factors. It is highly likely that such viral proteins mimic antiviral effectors induced in host cells upon viral infection.

To test this hypothesis, microarray data from mouse GM-CSF-induced dendritic cells stimulated with the Toll-like receptor 4 (TLR4) ligand lipopolysaccharide (LPS), the TLR2 ligand Pam3CSK4 (PAM), or Newcastle disease virus (NDV) were analyzed. Probes whose expression levels changed 3 fold or more, and 1.5 fold or less, were denoted “3-fold” and “1.5-fold” sets, respectively. The 3-fold set represented a set of genes induced or suppressed after stimulation, and thus directly or indirectly involved in innate immunity. In contrast, the 1.5-fold-set represents genes whose expression levels do not change upon stimulation. By using observed gene expression levels in this way we avoided the errors associated with incomplete functional annotations that would compromise statistics derived from database queries.

Each set corresponded to a list of affymetrix identifiers, which could be directly uploaded to MusVirus. A utility script was prepared that converted the resulting output to a table that could easily be imported into 3^rd^ party software.

We first computed the numbers of MusVirus hits for each gene in each set. As shown in Table 3, the 3-fold sets of LPS and NDV stimulation had significantly higher mean numbers of viral homologs than the corresponding 1.5-fold sets, whereas the 3-fold set of PAM stimulation had only marginal difference. Virus and LPS but not PAM induce an IRF-dependent antiviral signaling pathway [9]. Thus this result suggests that genes induced or reduced by antiviral signaling are preferentially mimicked by viruses.

This result next prompted us to ask if viruses are more likely to contain genes involved in the innate antiviral response rather than the adaptive immune response. To address this question microarray data from T cells stimulated via T cell receptors were examined. Two sets, 3-fold and 1.5-fold, were prepared as above, and checked for the numbers of hits in MusVirus. We found that the mean value of the T cell 3-fold set and that of the T cell 1.5-fold set were statistically comparable, indicating that viruses preferentially mimic genes involved in innate but not acquired immune response. When we examined the conserved domains present in the NDV 3-fold set that has 1 or more viral hits, we found that most (18%) belonged to the kinase family, consistent with the overview above. Other families consisted of proteins involved in ubiquitination (6%), transcription factors (5%), proteases (4%), helicases (3%) and phosphatases (2%).

**Table 3:** Mean numbers of hits in MusVirus of each gene sets.

Data set	3-fold	1.5-fold	***p***** value**
NDV	6.66	4.42	1.79 x 10^-3^
LPS	6.06	4.42	3.23 x 10^-4^
PAM	5.48	4.46	5.15 x 10^-2^
T cell	5.01	4.45	1.50 x 10^-1^

## Using MusVirus to search for proteins with known biochemical function

In this section we demonstrate how to search for proteins using keywords that map to specific biochemical functions. For the following we used mode-2 (low-complexity filtering turned on), as described in the Methods “Sequence Comparison” subsection. We focused on several functional classes of proteins that are known to affect host immune response. These included kinases, cytokines and their receptors, as well as proteins involved in ubiquitination.

### Kinases

Viruses often encode kinases to control viral growth by regulating viral gene expression, DNA synthesis, and tissue tropism. Searching with the entire “viral” DB for the word “kinase” resulted in 933 hits, 701 with mouse-virus homologs. By restricting the search to virus with “kinase” in the annotation field, 134 hits with homologs in mouse were found. By restricting the search to those with homologs, we expect to increase the chance of finding example where the acquired gene is used to exploit host signaling pathways for propagation or to subvert host immune responses.

**Figure 3:**
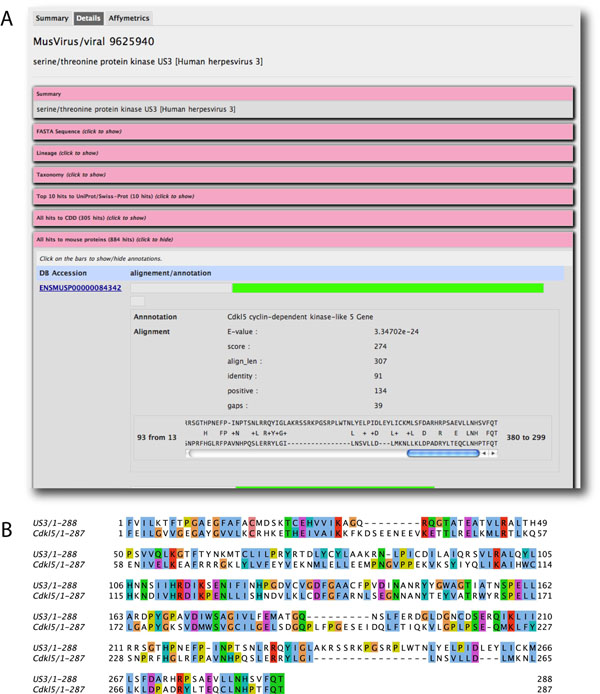
**Alignment between Human herpesvirus UL3 and mouse Cdkl5.** A) Screenshot for the aligned pair of sequences indicates the expectation value (E-value), as well as conserved residues. B) The alignment is shown again with amino acids colored by residue type.

Herpesviruses are known to possess many conserved kinases. Using the terms “kinase” AND “herpesvirus” resulted in 55 hits with mouse-virus homologs. If we restricted the search to the functional annotations, 23 hits with homologs were returned. Herpes simplex virus (HSV, Human herpesvirus 1) had 1 kinase hit US3 [10], which was found by the above search criteria in MusVirus. US3 (GenbankID 9629444) had 838 hits in MusVirus, most of which were other annotated kinases. The alignment between UL3 and the cyclin-dependent kinase-like 5 protein Cdkl5 is shown in figure 3. These homologs could not be identified by conventional BLAST searches, but were identified by using PSI-BLAST, employed in MusVirus. UL13 is reported to phosphorylate host EF-1delta, CK-IIbeta, and the large subunit of RNA polymerase II [10]. They are also reported to be substrates of kinases of the Cdk family [11], suggesting that viral and host kinases may share substrate specificity. These collectively indicate that MusVirus offers a convenient way to identify homologues, which could not easily be identified by typical sequence homology search engines, and enabled putative functional annotations, such as kinase substrate specificity, for some viral proteins.

### Cytokine receptors

**Figure 4:**
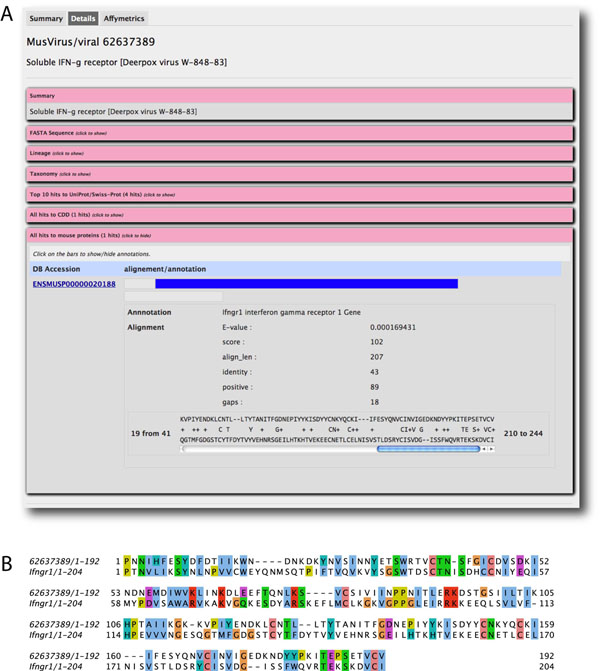
**Alignment between the soluble IFN-g receptor from Deerpox virus and the mouse interferon gamma receptor 1.** A) Screenshot for the aligned pair of sequences indicates the expectation value (E-value), as well as conserved residues. B) The alignment is shown again with amino acids colored by residue type.

An important signaling molecule targeted by viruses for molecular mimicry is tumor necrosis factor (TNF), which plays an essential role in orchestrating the response to pathogen invasion [12]. Viruses target TNF levels in various ways, including expression of soluble TNF-binding molecules that mimic TNF receptor (TNFRs) [13]. Performing the search “tumor necrosis factor receptor” over “CDD annotations” with the option “Virus Centered proteins” resulted in 30 hits were found with mouse-virus homology. The top hits include the CrmD protein in the Cowpox virus, which attenuates inflammatory responses in the mouse intestine [14], and the M-T2 protein from Myxoma virus, which blocks both TNF and virus-induced apoptosis [15]. Searching the viral-centered database with “require mouse-virus homology” disabled using the key words “interferon AND receptor'' yielded 18 viral protein hits. Among them, 16 viral proteins showed highest similarity to the mouse interferon gamma receptor 1 (Ensembl protein ID: ENSMUSP00000020188), but other proteins related to interferon or chemokine regulations were also found. The alignment between the soluble IFN-g receptor from Deerpox virus and the mouse interferon gamma receptor 1 is shown in figure 4. There may exist many other potential targets for viruses to interfere with the mouse cytokine regulatory system besides those annotated as interferon receptors.

### Cytokines

Viruses target cytokines and chemokines directly, through molecular mimicry, and also indirectly by targeting upstream or downstream regulators in cytokine/chemokine-mediated pathways [5]. Chemokines and cytokines are attractive targets for acquisition by viruses since they can affect a broad range of host defense responses, including intra and inter cellular signaling, chemotaxis, and cell death. They are also efficiently stored in DNA viral genomes due to their small size. The most studied viruses that encode homologs of host cytokines are herpesvirues. When the search “cytokine” NOT “receptor” was performed against the viral-centered database with “require mouse-virus homology” disabled, 10 hits were found, most of which were dsDNA viruses (herpes viruses, variola virus, Infectious spleen and kidney necrosis virus, etc.). The top hit was to a Putative CC-type chemokine U83 in Human herpesvirus 6B, which, is also found in the 6A strain and functions as a chemoattractant for monocytes [16].

### Ubiquitin-related proteins

Some viruses manipulate their hosts' ubiquitin system for their propagation [17]. Some ubiquitin-like proteins in viruses such as USP7 protect other viral proteins from ubiquitination in the host cell and suppress NF-κB signaling. Searching CDD annotations for viral proteins with the keyword ''ubiquitin'' identified 246 mouse-virus homolog hits, mostly to E3 ubiquitin ligases. Ubiquitin E3 ligase-like proteins are known to be involved in processes such as activation of viral and cellular genes, degradation of receptor molecules (e.g., MHC class I), as well as suppression of innate immunity. Among such ubiquitin E3 ligase-like proteins, 19 infected cell protein 0 (ICP0) from various herpesviruses were found in MusVirus and they were indeed homologous to many mouse proteins containing the RING finger motif.

Recently, a number of examples of viruses acquiring genes involved in host defense have been described [5-7,10,17,18]. Together, these cases studies provide crucial information about regulation of the animal immune system. The purpose of MusVirus is to provide a platform where researchers can systematically search for functionally relevant homologous relationships between animal virus and host genes. MusVirus was designed to operate on the level of protein domains in order to make biochemical functional interpretation of results as unambiguous as possible.

As the examples in this study show, MusVirus hits cover various functional classes of proteins exploited by viruses. In order to be useful as a tool for functional prediction and discovery, we included mapping between DNA microarray identifiers and mouse proteins. Our investigation of gene expression data in dendritic cells stimulated with several ligands (LPS, PAM3CSK4, and NDV) indicate that there is a significant difference in the number of host acquired genes differentially expressed during the innate immune response compared to genes whose expression levels do not change. Interestingly, we could classify such genes as belonging particularly to a viral (NDV and LPS 3-fold sets) as opposed to an antibacterial innate immune response (PAM 3-fold set). Analysis of gene expression in T cells indicates that viruses are more likely to acquire genes involved in innate immune responses than that in acquired immune response.

Although we have focused on mouse as a host species of animal viruses in this study, the present approach is readily applicable to other host species such as humans. By compiling databases analogous to MusVirus for various host species, it may help to elucidate universality as well as intricate diversity of viral infection, host defense mechanisms and their interplay. Indeed, the above hits to mouse kinases (Rrm1, Cdk6, Crkrs, EF-1delta, CK-IIbeta, and the large subunit of RNA polymerase II), and cytokine receptors (interferon gamma receptor 1) all had human orthologs.

## Conclusions

Collectively, MusVirus captures the preference of viruses to mimic proteins involved in acute antiviral immune responses presumably because viruses take advantage of these proteins to suppress host immune system for survival and dissemination. We believe that this database will benefit research in both immunology and virology by providing a vehicle for functional insights on various genes.

## Methods

### Data sources

40,732 mouse proteins were obtained from the Ensembl database (NCBIM37.56 release). The viral proteins were obtained from RefSeq. A list of the correspondence between viruses and their hosts were provided by the Database of the International Committee on Taxonomy of Viruses (ICTVdB). Proteins from viruses that infect Algae, Archaea, Bacteria, Fungi, or Plants as well as all phages were discarded. All other viral proteins, including those with un-annotated hosts, were retained. In total, there were 32,928 proteins from 1,190 viral species. We note that proteins from animal viruses whose host is not mouse were retained since their homologs may also provide valuable information about animal immune systems. There were 24 viral species whose host was identified as “mouse” (more precisely, their scientific names contained the words “mouse,” “murid” or “murine”). In this work, we refer to these 24 viruses as “mouse-infecting viruses.”

### Sequence comparison

We employed two different modes for finding homologs between mouse and viruses. In Mode-1, PSI-BLAST [19] was used for sequence similarity searches. Position-specific scoring matrices (PSSMs) were created for mouse and viral proteins by iterating PSI-BLAST (with low-complexity filter) three times against the UniRef90 [20] database with an e-value cutoff of 10^−3^ and with no limit for the number of displayed alignments (other parameters were set to default values). After a PSSM was made for each mouse (or viral) protein sequence, PSI-BLAST searches were conducted against viral (or mouse) proteins, UniProt/SwissProt, and CDD databases (only amino acid sequences, not PSSMs, were used for CDD). In the production run of PSI-BLAST, we turned off the low-complexity filter. Although this may introduce some false positives, we noticed that some meaningful hits were only found without low-complexity filtering. In Mode-2, we used BLAST with low-complexity filter for all the cases, except for the search against CDD where RPS-BLAST was used using the CDD PSSMs. In all BLAST/RPS-BLAST searches, the parameters were equivalent to those in Mode-1.

### Compilation of sequence comparison data

The results of the PSI-BLAST searches and annotations were compiled into a relational database. For mouse proteins, annotations of gene ontology, mouse genome initiative, affymetrix identifiers (mouse430 2 and mouse430a 2) and InterPro were extracted from Ensembl using the BioMart [21] interface. Predicted protein localizations were obtained from eSLDB [22]. For viral proteins, annotations of NCBI taxonomy were compiled together with host information from the ICTV database [23]. The sources of protein sequences and annotations is available from Table 4.

**Table 4:** Sources of protein sequences and annotations.

Resource	data type	data source
Mouse proteins	Protein sequence	Ensembl
Viral proteins	Protein sequence	RefSeq^2^
UniProt/SwissProt	Protein sequence	UniProt^3^
UniRef90	Protein sequence	UniProt^3^
CDD	Protein domains	NCBI^4^
NCBI Taxonomy	Taxonomy	NCBI^5^
eSLDB	Subcellular localization	eSLDB^6^
GO	Gene ontology	Gene Ontology^7^

^1^http://www.ensembl.org, ^2^http://www.ncbi.nlm.nih.gov/RefSeq/, ^3^http://www.uniprot.org, ^4^http://www.ncbi.nlm.nih.gov/Structure/cdd/cdd.shtml, ^5^http://www.ncbi.nlm.nih.gov/Taxonomy/taxonomyhome.html/, ^6^http://gpcr2.biocomp.unibo.it/esldb/, ^7^http://www.geneontology.org/

### Web interface

A web interface for the MusVirus database was constructed (http://imm.ifrec.osaka-u.ac.jp/musvirus/). The user can construct queries by searching over various fields in MusVirus. CDD keywords are useful for finding proteins that share particular conserved domains, in contrast to sharing only local homology. For mouse proteins, affymetrix IDs can also be used as input. The latter feature is useful for identifying the intersection between inducible genes and the corresponding proteins shared between mouse and virus. It is also possible to search by browsing a viral taxonomy tree, thus restricting the search to a particular subset of viruses.

### Microarray data processing and statistical analysis

Microarray data of gene expression in GM-CSF-induced bone marrow dendritic cells (GM-DC) stimulated with Newcastle disease virus was described previously [24]. Microarray data of gene expression in GM-DC stimulated with Pam3CSK4 or LPS [25], and in T cell stimulated with anti-CD3 antibody [26] were described and obtained from NCBI GEO (accession numbers GSE17721 and GSE12464, respectively). Robust multiarray expression values were calculated from the raw intensity data with using R and Bioconductor (http://www.bioconductor.org). Probes having 3 fold or more difference relative to unstimulated conditions were denoted as the “3-fold set”. Probes having 1.5 fold or less difference were denoted the “1.5-fold set”. For each probe, the number of hits in MusVirus were counted using “mode 2”, as described above, and then the number was divided by the number of proteins corresponding to the probe. The resulting number was used as the number of MusVirus hits for the probe. The Welch’s *t* test was then applied to these sets and *p* values were calculated using R.

## Competing interests

The authors declare that they have no competing interests.

## Authors’ contributions

OT conceived the general idea of the study; ARK carried out sequence comparisons and implemented the backend database; YK performed the statistical analyses on gene expression data; HD implemented the web interface; all authors analyzed the results, and wrote and approved the manuscript.

## Supplementary Material

Additional File 1 Tables of mouse-infecting virus proteins that have mouse homologs (c.f. Table 2). Click here for file
